# High Temperature Inhibits Ascorbate Recycling and Light Stimulation of the Ascorbate Pool in Tomato despite Increased Expression of Biosynthesis Genes

**DOI:** 10.1371/journal.pone.0084474

**Published:** 2013-12-19

**Authors:** Capucine Massot, Doriane Bancel, Félicie Lopez Lauri, Vincent Truffault, Pierre Baldet, Rebecca Stevens, Hélène Gautier

**Affiliations:** 1 INRA, UR 1115 Plantes et Système de cultures Horticoles, Avignon, France; 2 EA4279, Université d’Avignon, Avignon, France; 3 INRA, UMR 1332 Biologie du Fruit et Pathologie, France Université de Bordeaux, Bordeaux, France; 4 INRA, UR 1052, Génétique et Amélioration des Fruits et Légumes, Montfavet, France; Universidade Federal do Rio Grande do Sul, Brazil

## Abstract

Understanding how the fruit microclimate affects ascorbate (AsA) biosynthesis, oxidation and recycling is a great challenge in improving fruit nutritional quality. For this purpose, tomatoes at breaker stage were harvested and placed in controlled environment conditions at different temperatures (12, 17, 23, 27 and 31°C) and irradiance regimes (darkness or 150 µmol m^-2^ s^-1^). Fruit pericarp tissue was used to assay ascorbate, glutathione, enzymes related to oxidative stress and the AsA/glutathione cycle and follow the expression of genes coding for 5 enzymes of the AsA biosynthesis pathway (*GME*, *VTC2*, *GPP*, *L-GalDH*, *GLDH*). The AsA pool size in pericarp tissue was significantly higher under light at temperatures below 27°C. In addition, light promoted glutathione accumulation at low and high temperatures. At 12°C, increased AsA content was correlated with the enhanced expression of all genes of the biosynthesis pathway studied, combined with higher DHAR and MDHAR activities and increased enzymatic activities related to oxidative stress (CAT and APX). In contrast, at 31°C, MDHAR and GR activities were significantly reduced under light indicating that enzymes of the AsA/glutathione cycle may limit AsA recycling and pool size in fruit pericarp, despite enhanced expression of genes coding for AsA biosynthesis enzymes. In conclusion, this study confirms the important role of fruit microclimate in the regulation of fruit pericarp AsA content, as under oxidative conditions (12°C, light) total fruit pericarp AsA content increased up to 71%. Moreover, it reveals that light and temperature interact to regulate both AsA biosynthesis gene expression in tomato fruits and AsA oxidation and recycling.

## Introduction

Ascorbate or ascorbic acid (AsA) is an important micronutrient essential for plant growth [1,2] which plays major roles in the interaction of plants with the environment, pathogens and oxidizing agents [3–5]. Moreover, AsA is an essential antioxidant for humans. Besides the well-known role of vitamin C in preventing scurvy, AsA is involved in preventing various oxidative stress-related illnesses such as cancers, cardio-vascular diseases and aging [6–8]. Since humans have lost the ability to synthesize AsA, fruits and vegetables are the major source of dietary AsA for humans. Tomato (*Solanum lycopersicum* L.) is produced worldwide and year-round. AsA concentration in tomato fruit depends on the cultivar [9]. Owing to its large consumption, tomato fruit constitute an important source of AsA intake in the human diet. Fruit AsA content strongly varies with the pre- and post-harvest environment [10,11], and thus an improved understanding of the regulation of AsA accumulation in fruits is required. 

AsA pool size in fruit depends on its import (or the import of precursors) from leaves, its synthesis and recycling (reduction of the oxidized form) within the fruit and its export, as well as its degree of oxidation or degradation. The major AsA biosynthesis pathway in plants, namely the L-Galactose pathway [12], has been fully elucidated in *Arabidopsis* leaves [2]. It involves ten enzymatic steps starting from D-Glucose to L-AsA. The fifth and sixth steps, corresponding to GDP-D-mannose pyrophosphorylase (GMP) and GDP-D-mannose 3’,5’ epimerase (GME) respectively, are used to synthesize activated nucleotide sugars that are also precursors of cell wall polysaccharides and glycoproteins. The committed pathway starts with GDP-L-galactose phosphorylase (GGP) which is coded for by two genes: *VTC2* and *VTC5* [2,13,14]. Subsequently GPP (L-Galactose-1-P-phosphatase) and L-GalDH (L-galactose dehydrogenase) convert L-Galactose-1-phosphate to L-Galactono-1,4-lactone. Finally, GLDH (L-galactono-1,4-lactone dehydrogenase) converts L-Galactono-1,4-lactone to L-ascorbate. Interestingly, the genes coding for these enzymes are also expressed in fruits e.g. in apple [15] and tomato [1,16,17] indicating that fruits possess the ability to synthesize their own ascorbate. In addition, alternative pathways may also contribute to increasing the AsA pool size, but their contribution depends on species and developmental stages [18–20].

Once synthesized, AsA can rapidly be oxidized, as result of its antioxidant function, so that the recycling pathway also plays an important role in maintaining AsA levels and redox state in plant cells [5]. AsA is oxidized to monodehydroascorbate (MDHA) chemically or enzymatically by ascorbate peroxidase (APX) [21]. As a very unstable compound, two MDHA molecules can spontaneously disproportionate into AsA and dehydroascorbate (DHA) or be reduced by NAD(P)H dependant-monodehydroascorbate reductase (MDHAR) activity. DHA can further be reduced enzymatically to AsA by dehydroascorbate reductases (DHAR) using glutathione (GSH) as reducing substrate, and the oxidized glutathione (GSSG) thus produced can be reduced back to GSH by glutathione reductase (GR) activity. These reactions are known as the ascorbate-glutathione cycle (AsA-GSH) [22,23].

Finally, if not recycled to AsA, recent work has demonstrated that DHA can be oxidized/degraded to produce oxalate and threonate residues [24,25]. 

Tomato fruit AsA content varies with the season [26,27] probably due to light or temperature differences. Light has been shown to strongly influence AsA metabolism in photosynthetic tissues. Indeed, in leaves, light increases the expression level of genes and the corresponding activities involved in the AsA biosynthetic pathway [2,17,28,29] but also the activity of enzymes involved in the AsA recycling pathway [30,31]. Less is known about light regulation of AsA content in fruits. One study in apple fruit reported the role of light in regulating genes involved in AsA synthesis and recycling [15]. Similarly in mango fruit peel, total and reduced AsA content increased with fruit irradiance from shaded fruits to the sunny side of well exposed fruits [32]. In this study, increased AsA content was related to increased activities of some enzymes of the AsA/glutathione cycle, APX, MDHAR, and in some cases DHAR and GR. Recently, we showed that following 7 days of shading, decreased AsA content in red ripe fruits was related to the down-regulation of two AsA biosynthesis genes: *VTC2, GPP1* [17]. However, as light and temperature are often positively related, the relative role of temperature on AsA content remains unclear and has been rarely distinguished from the influence of light on growing on-vine tomatoes [33,34]. The lower fruit AsA content, which has been reported at high temperatures [35,36] could be related to increased AsA oxidation and degradation when the temperature increases. Thus, understanding how fruit AsA biosynthesis and recycling are affected by irradiance depending on temperature is still a challenge owing to the difficulty in separating these factors under natural conditions.

Moreover, in the context of climate change, elevated temperatures may affect fruit development and metabolism and consequently fruit yield and quality [37]. This study therefore aimed at understanding how elevated temperature combined with irradiance during fruit ripening will affect fruit ascorbate content.

Our objective was to study the regulatory mechanisms involved in AsA accumulation in fruit. In a previous study, we reported that the regulation of fruit AsA content was more highly dependent on the fruit microclimate than the leaf microclimate [38]. Therefore in the present work, the impact of light was studied at different temperatures during off-vine ripening of tomato fruits thus excluding any import to, or export from, the fruit. We investigated how temperature modulates the effect of light on AsA synthesis and recycling, as both contribute to increasing and maintaining AsA pool size, by quantifying transcript levels of AsA biosynthetic genes and by measuring AsA recycling activities in vitro.

## Materials and Methods

### Plant growth

The study was performed on cherry tomato *Solanum lycopersicum* L. ‘West Virginia 106’ (‘WVa 106’). Plants were grown in a glasshouse in Avignon (Southern France, 44°N). Plants with ﬁve growing leaves were transplanted on 31^st^ August 2009 into 5 L pots containing potting soil in a multispan Venlo-type greenhouse, N–S orientated. Plant nutrition, chemical pest and disease control were carried out in accordance with commercial practices. Water was supplied to the plants using a drip irrigation system to maintain 20–30 % drainage. Flowers were mechanically pollinated three times a week. All plant side shoots were removed as they appeared.

### Fruit sampling and treatments

Fruits were harvested at breaker stage (corresponding to 32 days after anthesis) at dawn and peduncle scars were covered with Terostat to limit water loss. This stage of development was chosen as shading fruits earlier on the vine had no effect on fruit AsA content at harvest, but shading fruit at the green mature stage and later significantly affected fruit AsA content at harvest (data not shown). Harvested fruit were weighed and external colour assayed. They were then split into three groups having similar mean size and colour: one was used to assay pericarp composition at harvest; another one was placed under continuous light for 56h and the last one in darkness for 56h to discriminate the impact of light on fruit composition. This distribution of fruits was repeated for each temperature tested. External colour was characterized near the pistil scar by a Minolta Chroma meter (CR 300, Minolta, France SA) using the Hunter color coordinates *L*, *a*, and *b*; (*L* lightness, *a* ranging from green to red, *b* ranging from blue to yellow). Fruits were then placed in two growth cabinets (Sanyo, MLR-351H) under controlled temperatures either in darkness D= 0 µmol m^-2^ s^-1^) or under light (L= 150 µmol m^-2^ s^-1^). Successive experiments were undertaken to determine the impact of irradiance at 31°C, 27°C, 23°C, 17°C and 12°C. To adjust fruit temperature between light and darkness, additional fruits at breaker stage were harvested and fruit temperature was measured every minute with very ﬁne thermocouples (0.2 mm copper–constantan). Thermocouples were inserted to a depth of 2 mm into the upper exposed side of fruits and temperature measurements were averaged and recorded every 30 min by a delta logger (Delta-T DL2e, Delta-T Devices Ltd, Cambridge, UK). The air temperature in the cabinet with light was slightly decreased (-0.5°) in order to obtain similar fruit temperatures. Vapor pressure deficit was identical for all temperatures tested (0.56 kPa). 

### Fruit measurements

After 56h, 4 replicates of three fruits per treatment were collected and evaluated individually for physical traits, including fresh weight (FW) and external colour. Fruits were cut on ice, seeds and gel were discarded, and pericarp tissue was frozen in liquid nitrogen and stored at -80°C before grinding in liquid nitrogen.

### Ascorbate and glutathione assays

Assays of total, reduced and oxidized AsA content were carried out on ground powder conserved at -80°C as previously described [39]. Total ascorbate (reduced + oxidised forms) was measured by mixing the sample with 5mM DTT, to reduce dehydroascorbate, prior to the assay. The specificity of the assay has been checked by comparison with other known methods [39] and by using ascorbate oxidase to remove all ascorbate in order to detect background absorbance [38,40]. Assays of total glutathione were measured according to a previously described protocol adapted as a microplate assay [41–43]. The assay was based on the reaction of reduced glutathione with 5,5’-dithiobis-2-nitrobenzoic acid (DTNB) producing 5-thio-2-nitrobenzoic acid (TNB) that absorbs at 405 nm. Extractions were carried out in duplicate in 5 % sulfosalicylic acid. To measure total glutathione, 10 µl of extract was mixed with 150 µl of a mix containing 0.2 mU/µl of glutathione reductase and 0.04 mg/ml DTNB in 120 mM phosphate buffer pH7 with 5.7 mM EDTA, the reaction was started by the addition of 5 µl of 2 mM NADPH and the increase in absorbance at 405 nm was measured over a period of 10 minutes and compared to standard concentration range from 0 to 0.5 nmol of reduced glutathione (GSH) per well. Each extract was measured in duplicate.

### RNA extraction and qPCR analysis

RNA extractions were performed as previously described [17] from the pericarp tissue of fruits placed at 12°C, 23 and 32°C for 56h. To eliminate DNA contamination, 15µg of total RNA was treated with RQ1 RNAse free DNAse (Promega). Reverse transcription was performed with 5µg of DNA free RNA, treated with RNasin (Promega) to inhibit RNAse activity, using oligo (dT)_21_ (10µM) and SuperScript® II Reverse Transcriptase (Invitrogen) according to manufacturer’s instructions. The cDNA obtained was diluted 5 fold in RNAse-free water and 2µL aliquots were stored before use. Quantitative real-time PCR analyses were performed with the Stratagene Mx3005P® thermocycler (Stratagene, Cedar Creek, TX) using the Gotaq® qPCR Master Mix (Promega) according to manufacturer’s instructions in a reaction volume of 24.5µL. Expression analyses were performed for the six final genes of the AsA biosynthesis pathway (from GME to GLDH). As *VTC5* transcript levels were very low in fruits, it was excluded from this study. We analyzed four GDP-D-mannose pyrophosphorylase genes [*SlGMP1* (Solyc03g113790), *SlGMP2* (Solyc06g051270), *SlGMP3* (Solyc03g096730) and *SlGMP4* (Solyc09g011220)], two GDP-D-mannose-3’,5’-epimerase [*SlGME1* (Solyc01g097340), *SlGME2* (Solyc09g082990)], one of the two GDP-L-galactose phosphorylase genes [*SlVTC2* (Solyc06g073320)], two L-galactose-1-phosphate phosphatase genes [*SlGPP1* (Solyc04g014800), *SlGPP2* (Solyc11g012410)], a L-galactose dehydrogenase gene [*SlGalDH* (Solyc01g106450)] and a L-galactono-1,4-lactone dehydrogenase gene [*SlGLDH* (Solyc10g079470)]. For each reaction, two technical replicates were run. Relative gene expression was calculated by the 2^-ΔΔC^_T_ method using PCR efficiencies of the target and endogenous reference genes [44]. [*SlActin* (Solyc03g078400) and *SlEIF-4A-2* (Solyc12g095990)] were used as internal controls for calculation of a normalization factor using the Genorm method. Primer sequences are detailed in Table S1.

### Assay of enzyme activities

Protein extraction was performed according to the method described by Murshed et al. [45]. Briefly, 125 mg of frozen fruit powder was homogenized in 1 mL of 50mM MES/KOH buffer (pH 6) containing 40 mM KCl, 2 mM CaCl_2_ and 1 mM AsA for 3 min at room temperature. Extracts were centrifuged at 4°C for 15 min at 16000g and the supernatants were analyzed immediately for enzyme activities. 

APX, MDHAR, DHAR and GR activities were measured according to the method described by Murshed et al. [45] slightly modified as follows. Assays were carried out at 28°C using a microplate sprectrophotometer Infinite 200® (Tecan, Switzerland) in an UV-microplate (Hellma) for APX and DHAR and in a plastic microplate (Nunc) for MDHAR and GR. To calculate enzyme activity, absorbance was measured for 5 min before and 10 min after adding the substrate. The reaction rates (initial and after substrate addition) were calculated on linear slopes, for 2 to 4 min depending on the enzyme. Enzyme activity was corrected taking into account the initial reaction rate and the blank reaction.

APX activity was measured in a reaction mixture containing 165 µL of 50mM potassium phosphate buffer (pH 7.0), 0.25 mM AsA, 25µL of enzymatic extract and 10µL of 50 mM H_2_O_2_ to start the reaction. Activity was determined by measuring the disappearance of AsA at 290 nm using an extinction coefficient of 2.86 mM^-1^ cm^-1^.

MDHAR activity was measured in a reaction mixture containing 175 µL of 100 mM Hepes buffer (pH 7.6), 2.5 mM AsA, 0.25 mM NADH, 20µL of enzymatic extract and 5µL of 17 U/mL ascorbate oxidase to start the reaction. Activity was determined by measuring the disappearance of NADH at 340 nm using an extinction coefficient of 6.22 mM^-1^ cm^-1^.

DHAR activity was measured in a reaction mixture containing 185 µL of 50 mM Hepes buffer (pH 7), 0.1 mM EDTA, 2.5 mM reduced glutathione, 15µL of enzymatic extract and 15µL of 2.7 mM dehydroascorbic acid to start the reaction. Activity was determined by measuring the appearance of AsA at 265 nm using an extinction coefficient of 14 mM^-1^ cm^-1^.

GR activity was measured in a reaction mixture containing 165 µL of 50 mM Hepes buffer (pH 8), 0.5 mM EDTA, 0.25 mM NADPH, 30µL of enzymatic extract and 5µL of 20 mM oxidized glutathione to start the reaction. Activity was determined by measuring the disappearance of NADPH at 340 nm using an extinction coefficient of 6.22 mM^-1^ cm^-1^.

We also measured catalase (CAT) activity, an enzyme scavenging H_2_O_2_ that may neutralize an excessive production of reactive oxygen species produced during oxidative stress. CAT activity was measured using a method adapted from that of Aebi [46] in a reaction mixture containing 170 µL of 50 mM phosphate buffer (pH 7), 20 µL of enzymatic extract and 10 µL of 150 mM H_2_O_2_. Activity was determined by measuring the disappearance of H_2_O_2_ at 240 nm using an extinction coefficient of 43.6 M^-1^ cm^-1^.

### Statistical analysis

Statistical differences in metabolite concentrations, enzyme activities and gene expression were determined by analysis of variance, considering two factors ‘temperature’ and ‘light’ and their interaction. Mean separation was carried out using a Tukey test at 5 %. All statistical analyses were performed with the statistical software XLStat (Addinsoft, France).

## Results

### Fruit pericarp ascorbate and glutathione contents depend on interactions between light and temperature

**Figure 1:**
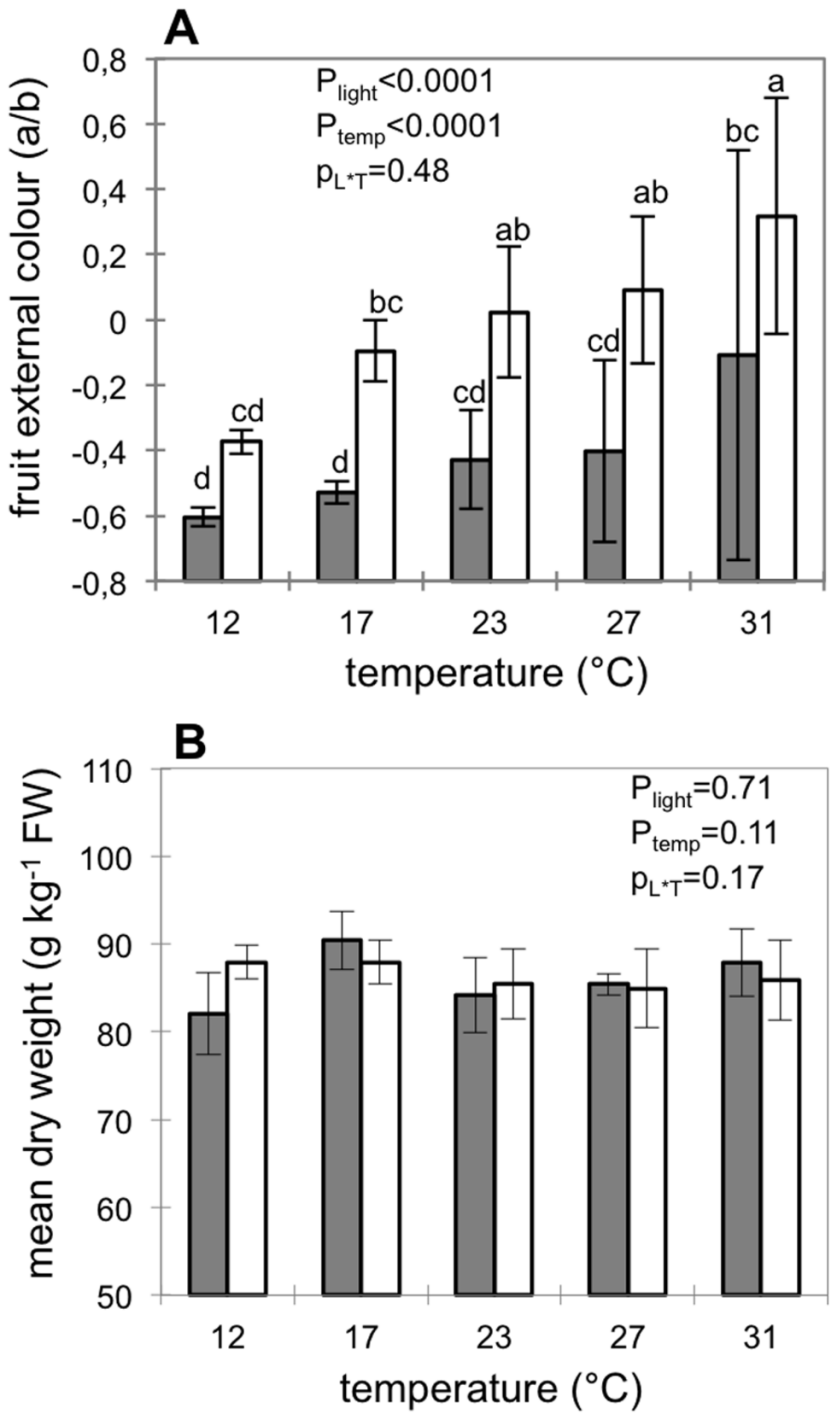
Difference in external colour and dry weight. External colour (A) and pericarp dry weight content (B) of fruits placed for 56h in growth cabinet under darkness (grey bars) or light (open bars) at different fruit temperatures. Data are means ±SD of 12 replicates (corresponding to 12 fruits). P-values of the two way ANOVA considering the factors light (p_light_) and temperature (p_temp_) and their interactions (p_L*T_) were given in the tables. Different letters indicate significant differences (Tukey test, p=0.05).

Changes in external fruit color were assessed from the a/b ratio that was previously shown to linearly increase during tomato fruit ripening [35,47]. In our study, we observed higher a/b ratios for fruits kept 56 hours under light compared to those kept in the darkness, which confirms that fruit picked at breaker stage were still able to ripen and synthesize lycopene under light (Figure 1A). 

**Figure 2:**
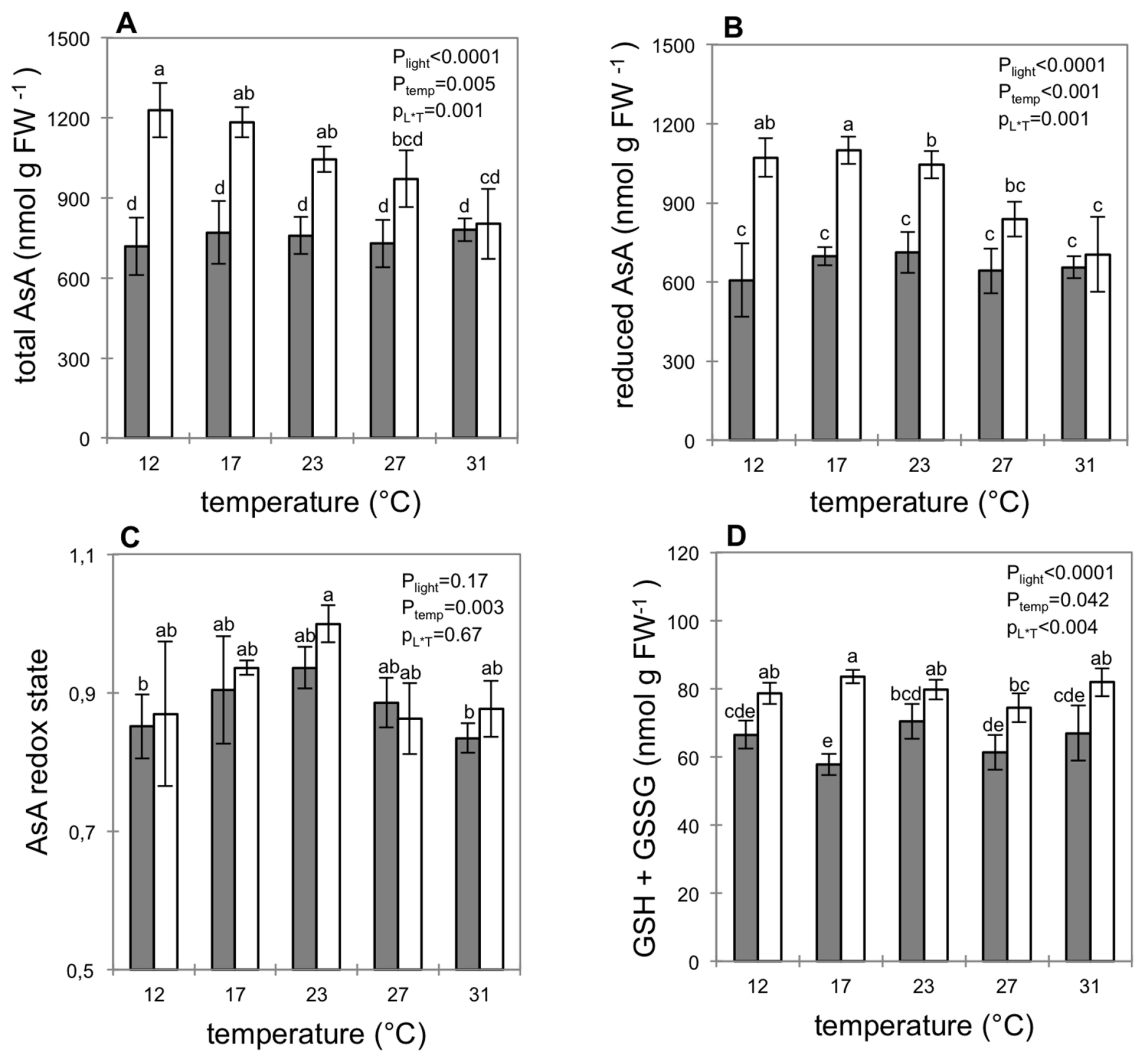
Total AsA(A), reduced AsA (B), AsA redox ratio (C) and total glutathione (D) in pericarp of fruits placed for 56h in growth cabinet under darkness (grey bars) or light (open bars) at different fruit temperatures. Data expressed in nmol g^-1^ fresh weight are means ±SD of 4 replicates (corresponding to 12 fruits). P-values of the two way ANOVA considering the factors light (p_light_) and temperature (p_temp_) and their interactions (p_L*T_) were given in the inset. Different letters indicate significant differences (Tukey test, P=0.05).

At harvest, total AsA content in fruit pericarp was not significantly different between successive experiments (p=0.19) and was around 820 nmol AsA g^-1^ FW (data not shown). Fruit pericarp dry weight content was not affected by temperature or irradiance (Figure 1B), and consequently, we chose to express all data per fresh weight. Keeping fruits for 56 hours in darkness did not modify total or reduced AsA content in fruit pericarp whatever the temperature tested (data not shown). In contrast, fruits kept 56h under continuous light had higher pericarp AsA content compared to fruits at harvest or fruits kept 56h in darkness (Figure 2A, 2B). This increased AsA content with light was dependent on temperature and decreased as temperature increased, so that at 27°C or 31°C there was no more effect of light on total or reduced AsA content. Although light triggered differences in AsA content, the redox ratio (AsA/(AsA+DHA) remained similar and was not significantly different from the redox ratio at harvest suggesting that after harvest, changes in the light environment do not affect fruit AsA redox state (Figure 2C). Whatever the temperature, glutathione content in fruit pericarp increased for fruit kept in the light compared to darkness (Figure 2D). Light triggered an increase in the glutathione pool either at low (12°C and 17°C) or high temperature (27°C and 31°C) but there was no significant effect of light on glutathione pool size in fruit pericarp at 23°C. To understand the mechanisms responsible for higher AsA accumulation under light, we measured the transcript level of genes involved in the major AsA biosynthesis pathway and the enzymatic activities of the AsA/GSH cycle.

### Light and temperature interact to modify transcript levels of AsA biosynthesis genes

**Figure 3:**
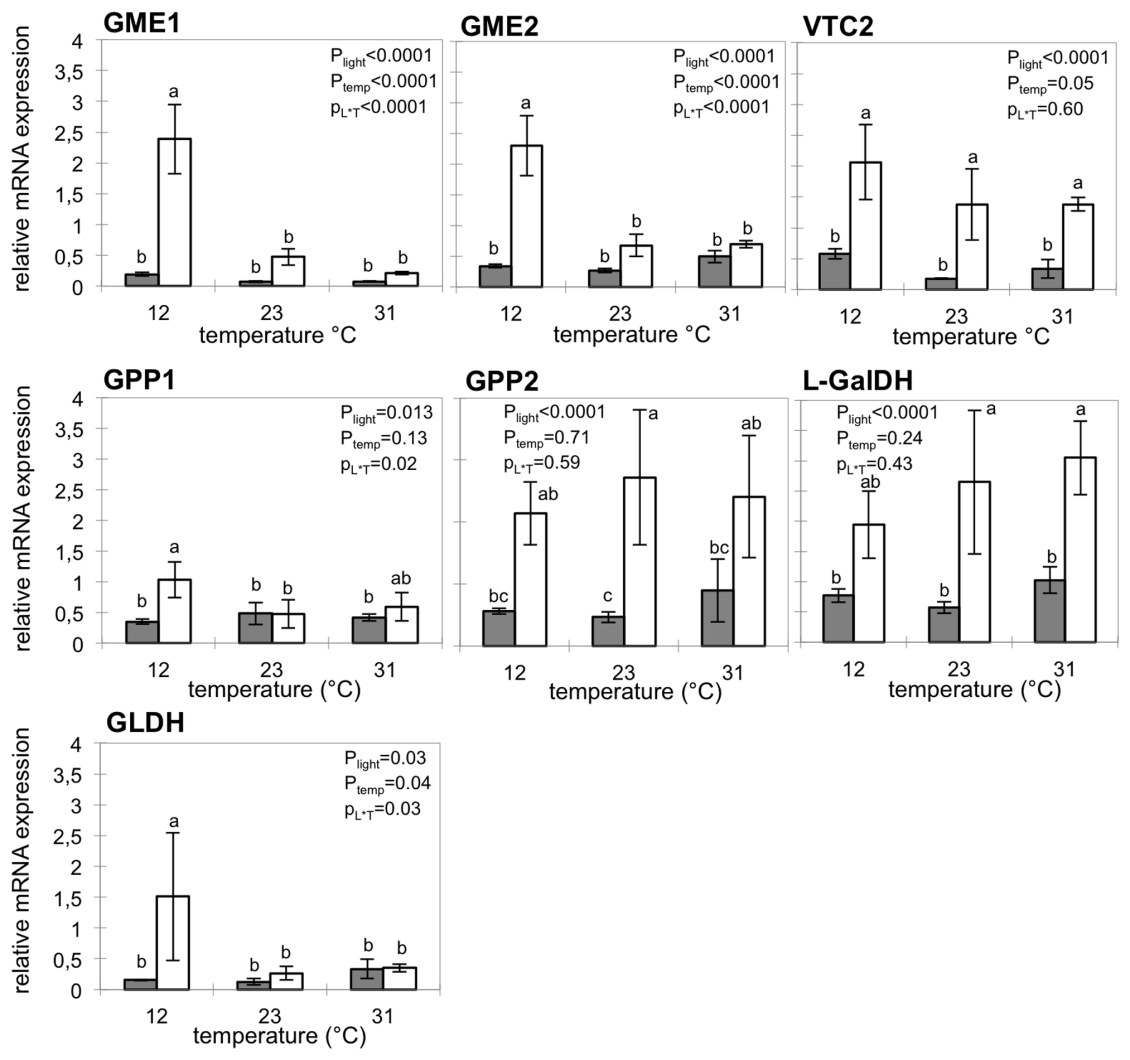
Transcript levels of genes of the AsA biosynthesis pathway. Data are the mean value ±SD of four replicates. Legend is similar to Figure 2.

In darkness, the transcript level of genes involved in the five final steps of AsA synthesis was low whatever the temperature, indicating that these genes were weakly expressed in darkness (Figure 3). Under light, the transcript level increased compared to darkness. However, we observed two different response patterns: *VTC2*, *GPP2* and *L-GalDH* transcript were two fold more expressed under light whatever the temperature experiment, whereas for the two *GME* genes, *GPP1* and *GLDH* transcript were more expressed under light at 12°C only (Figure 3). 

### Light and temperature interact to inhibit or enhance enzymatic activities related to AsA oxidation and recycling

**Figure 4:**
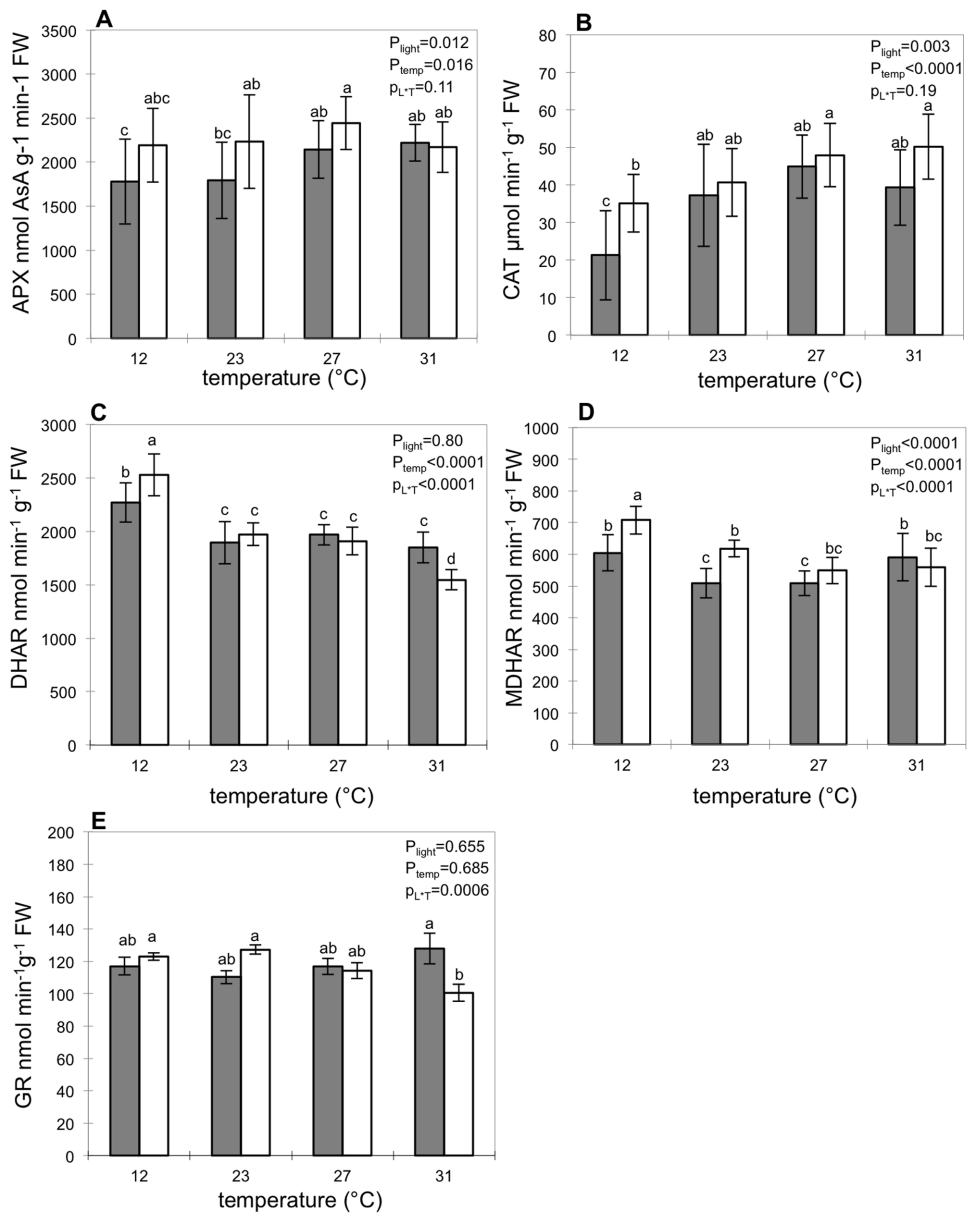
Enzymatic activities of the ASA/GSH cycle related to ASA recycling. Data are the mean value ±SD of four replicates. Legend is similar to Figure 2.

APX activity tended to be higher under light compared to darkness but no significant differences were found at each temperature tested (Figure 4A). Moreover APX activity increased significantly under darkness, while catalase activity increased in both darkness and under light (Figure 4A, 4B). Catalase activity was only significantly higher for fruits under light at 12°C, suggesting its requirement for detoxifying H_2_O_2_ under light at low temperature. At 12°C, the activities of DHAR and MDHAR were higher for fruits kept in the light compared to fruits kept in darkness (Figure 4C, 4D) and GR activity did not differ (Figure 4E). At 23°C, MDHAR was also more active in fruits kept in the light, whereas DHAR activity did not change. There was no significant difference in enzymatic activities between fruits kept in the light or darkness at 27°C, but at 31°C, both GR and DHAR activities were reduced in pericarp of fruits kept in the light.

## Discussion

### Light promotes the expression of AsA biosynthesis genes in fruit pericarp

The pericarp of fruits kept in the light during off-vine ripening contained up to 77% more reduced AsA than that of fruits stored in the dark. This confirms previous studies in tomato or blackcurrant indicating that fruit AsA content mostly depends on the importance of light reaching the fruit itself [38,48–50]. In our study, increasing fruit pericarp AsA content under light was combined with increased expression of at least three genes (*VTC2*, *GPP2* and *L-GalDH*) of the AsA biosynthetic pathway. In apple fruit skin, changes in AsA content have been related to light stimulation of the expression of the last two genes of the AsA biosynthetic pathway, *L-GalDH* and *GLDH* (other genes were not studied in [15]). In another study, shading of red ripe tomato during on-vine ripening also reported decreased fruit pericarp AsA content as well as *VTC2* and *GPP1* transcript levels [17]. The absence of light stimulation of *L-GalDH* may be related to the fruit developmental stage when light treatments were applied (red ripe stage in [17] compared to breaker stage in the present study), as *L-GalDH* expression decreases during fruit ripening and is very low in the late stage of fruit development [16]. Both studies on tomato (using off-vine and on-vine fruits) reported the light-controlled expression of genes encoding GGP and GPP which confirms the interest of putting fruits in controlled environment to modulate light and temperature independently, aiming at studying their respective effects on AsA metabolism. 

### Possible involvement of other light-regulated steps in AsA biosynthesis

At 23°C, AsA content in pericarp was enhanced under light but *GLDH* expression was not light affected. This may indicate that under moderate temperature, the final enzyme of the biosynthetic pathway is not limiting compared to *VTC2*, *GPP* or *L-GaLDH*. Previous studies have indeed reported that *GLDH* expression and activities were not limiting in tomato and kiwi fruits [1,51]. In addition post-transcriptional regulation of AsA biosynthesis may also occur; indeed, according to Leferink et al. [52], the light-regulation of AsA biosynthesis may be achieved by the direct modulation of GLDH activity. We cannot also exclude the possible involvement of alternative biosynthesis pathways that could be activated or inhibited depending on fruit microclimate [53]. Indeed, Badejo et al. [18], reported that the alternative D-galacturonate pathway contributes to ascorbate pool in microtom ripening fruits, in agreement with what was previously reported in strawberry fruits [20]. On the other hand, Ioannidi et al. [16] found that the expression of genes coding for alternative pathways in tomato fruit ‘Ailsa Craig’ decreased during fruit ripening. Moreover, these genes were not activated by heat stress but may be activated by cold stress, indicating that alternative pathways may contribute to AsA biosynthesis depending on genotypes, fruit developmental stage and abiotic stresses.

### Light and low temperature interact to promote AsA biosynthesis genes and AsA recycling

**Figure 5:**
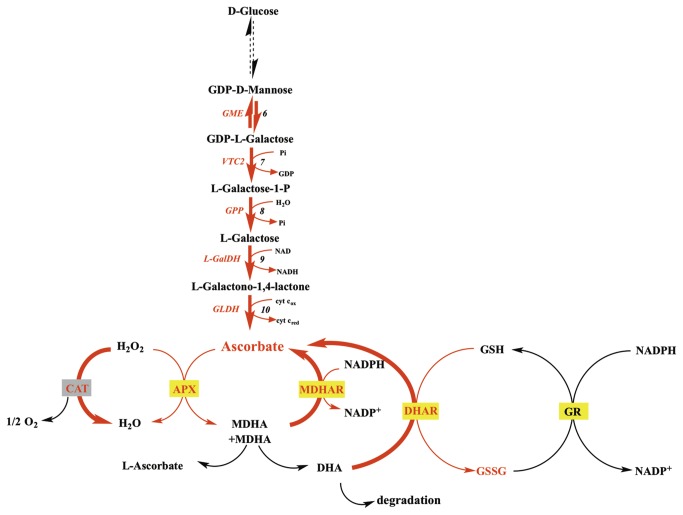
Differences in AsA regulation at 12°C in pericarp of fruits kept 56h in darkness compared to light. A red or blue color indicates a respectively higher or lower number of transcripts or enzymatic activity assessed by Tukey test (P=0.05). We investigated the impact on AsA synthesis by quantifying transcript levels of AsA biosynthetic genes and AsA recycling by measuring recycling enzyme activity in vitro.

**Figure 6:**
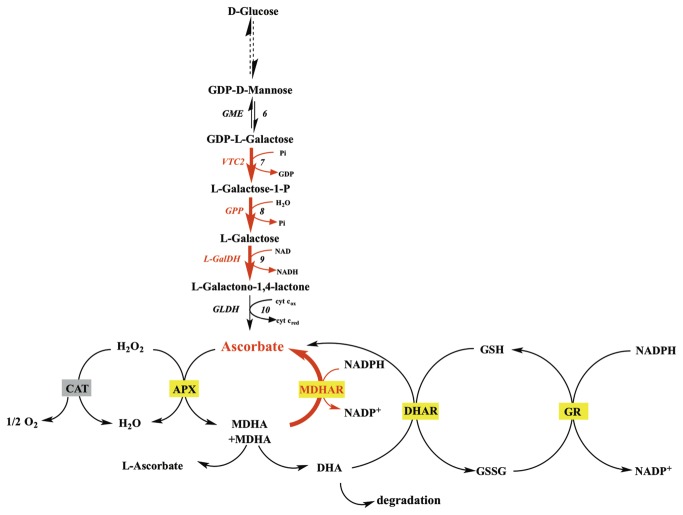
Differences in AsA regulation at 23°C in pericarp of fruits kept 56h in darkness compared to light. Legend is similar to Figure 5.

**Figure 7:**
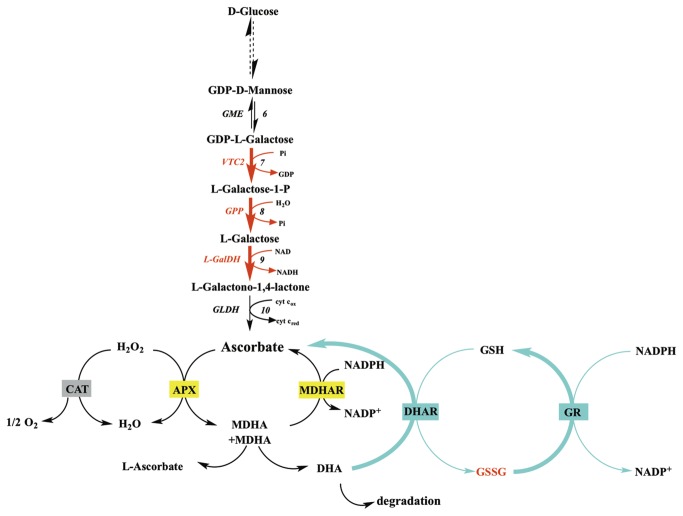
Differences in AsA regulation at 31°C in pericarp of fruits kept 56h in darkness compared to light. Legend is similar to Figure 5.

The present study also reveals interactions between light and temperature on AsA biosynthesis related genes in fruit pericarp. At 12°C, light triggered the enhanced expression of genes coding for GME and GLDH in addition to what was observed at 23°C or 32°C (Figures 5, 6, 7). At 12°C, light also increased the activity of antioxidant enzymes (CAT), and of AsA recycling enzymes (DHAR and MDHAR), leading to higher fruit pericarp AsA content (Figure 5). The assay of AsA/GSH enzymes showed higher APX and MDHAR activities with light exposure at 23°C and 12°C as previously shown in tomato fruits and the skin of apple fruits [15,54]. These results suggest increased use and recycling of AsA under light that could be explained by the role of AsA in photoprotection [55]. 

### Light up-regulation of AsA recycling decreases when temperature increases and is inhibited at 31°C

It was previously shown in grape fruits that fruit AsA content might be linked to transient changes in the expression of genes related to AsA biosynthesis (*GLDH*) but also to increased recycling (*MDHAR*) [56]. Therefore the simultaneous study of the impact of light on AsA biosynthesis, oxidation and recycling seems necessary in order to understand light regulation of AsA synthesis. In our study, at 31°C, it is interesting to note that enhancing the expression of *VTC2, GPP2* and *L-GalDH* was not sufficient to increase AsA content in fruit pericarp (Figure 7). At this temperature, we observe that light did not increase APX and MDHAR activities and even decreased DHAR and GR activities compared to darkness. The absence of light promotion of APX, MDHAR, DHAR and GR activities at 31°C (Figure 4) may be related to the inhibition of the AsA/glutathione cycle at high temperatures as previously reported in plants of tomato by Rivero et al. [57] at 35°C compared to 25°C. Several studies have stressed the role of AsA recycling pathway in maintaining AsA pool size and redox state [5]; changes in AsA recycling activities will impact plant growth and development, as well as responses to abiotic and biotic stresses. 

At 31°C, AsA oxidation and degradation may be enhanced as a consequence of lower recycling and it would be interesting to assay AsA degradation products, namely oxalate and threonate in tomato to estimate if increased temperature also increases AsA degradation. We noticed that at 31°C, light promotes glutathione synthesis even at high temperature. Thus, despite the absence of light promotion of AsA pool size, glutathione pool size was increased at high temperature, which may help the plant to cope with oxidative stress. 

Moreover, we observed that CAT activity increased in tomato fruit with temperature. Similarly, in tomato leaves, increasing temperature has been shown to trigger oxidative stress and to enhance CAT and APX activities [58,59]. In cucumber fruit, increasing the growing temperature from 27 to 32°C also promoted CAT activity [60]. As CAT helps to scavenge H_2_O_2_, this suggests a need to detoxify H_2_O_2_ probably due to oxidative stress. In agreement with this hypothesis, increasing temperature has been reported to trigger oxidative stress in fruits [1,16,54,61]. It might also be linked to the impact of temperature in enhancing ripening which is associated with increased oxidative stress in fruit [62].

## Conclusions

In conclusion, the present data give information on the way light and temperature interact to regulate fruit pericarp ascorbate content. We observed that high temperature triggers the inhibition of enzymatic activities of the AsA/glutathione cycle, limiting AsA regeneration and AsA pool size. Low temperature increased light-promotion of AsA biosynthesis transcription, giving clues about how to increase fruit AsA content during fruit ripening. In addition to the genetic control of the light-to-dark degradation of *VTC1* reported by Wang et al. [63] in Arabidopsis leaves, this study reveals the importance of temperature which might also affect the dark-to-light transcription of AsA biosynthesis genes. Considering the important role of AsA recycling in modulating AsA pool size, it seems necessary to better characterize ascorbate turn-over in fruits in different genotypes but also in different environments.

## Supporting Information

Table S1 **Sets of PCR primers used to amplify specific regions of ascorbate-related genes.**
(PDF)
