# Supplementary material for: High Temperature Inhibits Ascorbate Recycling and Light Stimulation of the Ascorbate Pool in Tomato despite Increased Expression of Biosynthesis Genes
Source: PLoS One. 2013 Dec 19;8(12):e84474. doi: 10.1371/journal.pone.0084474 (PMC3868655; doi:10.1371/journal.pone.0084474)
Supplement: Table S1 — Sets of PCR primers used to amplify specific regions of ascorbate-related genes. (PDF) [file pone.0084474.s001.pdf]

Table S1. Sets of PCR primers used to amplify specific regions of ascorbate-related genes

Name		Primer sequence 5' → 3'	Accession number
GMP1F	Forward	CATTGTCCTGCCAAACAAGA	Solyc03g113790
GMP1R	Reverse	AAAGGGCACTGCCAAATTC	
GMP2F	Forward	CCCACAAGGAGATCAAATC	Solyc06g051270
GMP2R	Reverse	TGAAACTGAAAACCAACCAAA	
GMP3F	Forward	TGGTTGGTTTCCAGTTCTCC	Solyc03g096730
GMP3R	Reverse	CAAAAATTACACATTTCCCAGTCTT	
GMP4F	Forward	TTCTCCGTCGCCATTTTTAC	Solyc09g011220
GMP4R	Reverse	CAGCCTTGTTCCAAAACCTC	
GME1F	Forward	ATTGGAAGCCAATCCATCTG	Solyc01g097340
GME1R	Reverse	AAACCAAGAAACGCCAACAA	
GME2F	Forward	TTGTTCGCGTGGAATGTTTA	Solyc09g082990
GME2R	Reverse	GTAGGTGCAATGAGGGGATG	
GGP1F	Forward	AAGGCAAAACCATGTTCAATG	Solyc06g073320
GGP1R	Reverse	ATGGGTCATTTCACACACCA	
GPP1F	Forward	TAGCCGCTACAAACCCTCAT	Solyc04g014800
GPP1R	Reverse	TCCGCTTTCCATCTCCTATG	
GPP2F	Forward	GGCCTTGAATGAATAAGAATGA	Solyc11g012410
GPP2R	Reverse	GCACATCGCGCAAAACTAT	
GalDHF	Forward	TGTTTGTCAGTTCAACGAGGTC	Solyc01g106450
GalDHR	Reverse	TTGTTTTAGATGTCCAAGTGCAA	
GLDHF	Forward	TTGCGAGGAAATTTTTGTCC	Solyc10g079470
GLDHR	Reverse	CTTGAAAAATGGCCTCAAGC	
ActinF	Forward	GGACTCTGGTGATGGTGTTAG	Solyc03g078400
ActinR	Reverse	CCGTTCAGCAGTAGTGGTG	
EIF-4A-2F	Forward	GATCAGCGTATCCTTCAGAG	Solyc12g095990
EIF-4A-2R	Reverse	GGCATTGTAGCAGAGAAAAC	
